# Higher Branched-Chain Amino Acid Intake Is Associated with Handgrip Strength among Korean Older Adults

**DOI:** 10.3390/nu13051522

**Published:** 2021-04-30

**Authors:** Seonghee Park, Minjeong Chae, Hyoungsu Park, Kyong Park

**Affiliations:** 1 Department of Food and Nutrition, Yeungnam University, Gyeongsan 38541, Gyeongbuk, Korea; seongheepark@ynu.ac.kr; 2 Nutrition and Functional Food Research Division, National Institute of Food and Drug Safety Evaluation, Cheongju 28159, Chungbuk, Korea; chaeminjeong@ynu.ac.kr; 3 Health & Nutrition R&D Group, Maeil Dairies Co., Ltd., Pyeongtaek 17714, Gyeonggi, Korea; parkhs@maeil.com

**Keywords:** branched-chain amino acids, leucine, dietary intake, older adults, handgrip strength

## Abstract

Sarcopenia is a disease of old age characterized by decreased muscle mass and strength. Branched-chain amino acids (BCAAs) promote muscle mass synthesis and increase muscle strength. We aimed to develop a dietary amino acid database and to examine the association between BCAA intake and handgrip strength in Korean older adults. Data from the Korea National Health and Nutrition Examination Survey 2014–2018 were used. Overall, 4852 participants aged ≥65 years were included in the study. Demographic, lifestyle, and health data were obtained through interviews and questionnaires. The 24-h recall method was used to assess dietary intake. An amino acid database was established using the 9th revision of the Korean Standard Food Composition Table. The mean handgrip strength was estimated from triplicate measurements obtained using the dominant hand. Multivariable linear regression analysis was performed to assess the association between BCAA intake and handgrip strength. Grains greatly contributed to amino acid intake; however, a significant negative association was observed between handgrip strength and increased BCAA intake through grains. In the fully adjusted model, leucine intake and handgrip strength showed a positive association. Thus, consuming BCAAs (especially leucine) via a variety of food sources can help maintain muscle health in older people.

## 1. Introduction

Aging is a phenomenon that reduces the ability to adapt to the external stress and the environment over time, resulting in a decreased physiological functional capacity and increased susceptibility to diseases [1]. During aging, numerous changes in body composition occur as muscles weaken and body fat levels increase [2], and the consequent decrease in muscle mass and muscle strength is known as sarcopenia [3]. Loss of skeletal muscle mass begins gradually at the age of 50 years and accelerates after the age of 70 years, resulting in an approximately 15% loss in skeletal muscle mass over 10 years [4]. Sarcopenia has been shown to increase the risk of falls and fractures [5,6], causing a reduction in physical activity [7] and ultimately leading to premature death [8]. The older population in Korea is expected to increase sharply from 12.8% in 2017 to 46.5% in 2067 [9]. Therefore, the prevalence of sarcopenia in older people will continue to increase, and the clinical and economic burdens are expected to gradually rise [10].

Sarcopenia can be defined based on the measurement of muscle mass and strength levels [3]. In particular, handgrip strength has been used to estimate muscle strength and can predict clinical prognosis more efficiently than muscle mass [11,12]. Previous studies have shown that hospitalized older patients with higher handgrip strength had a significantly shorter hospital stay and showed a lower frequency of re-hospitalization or risk of death [13], while the risk of cardiovascular disease-related death significantly increased as grip strength decreased by 5 kg [14]. Furthermore, a systematic review of 45 published papers showed that low handgrip strength was related to the risk of developing a disability and premature death [15], while another study revealed that muscle strength had a stronger correlation with physical disability or low physical performance and showed a better predictive value for these than muscle mass did [16].

Dietary habits can affect an individual’s muscle conditions. Dietary intake of amino acids and the intake of nutraceuticals, especially branched-chain amino acids (BCAAs) including leucine, isoleucine, and valine, are known to promote muscle protein synthesis and increase muscle strength as well as tendon health [17,18,19,20]. Several previous studies have examined the association between BCAA intake and muscle strength [21,22,23,24,25,26]; however, most of these studies have reported on the short-term effects of amino acid supplementation through clinical trials [21,22,23,24]. Furthermore, few community-based epidemiological studies have evaluated the association between habitual dietary BCAA intake and muscle strength in the Asian older population, particularly in South Korea, where the diet entails relatively low total energy intake and a large dependency on rice [27,28].

Therefore, this study aimed to determine the habitual dietary amino acid intake levels in Korean older adults aged ≥65 years utilizing data from the Korea National Health and Nutrition Examination Survey (KNHANES) and to analyze the association between the intake of BCAAs and handgrip strength.

## 2. Materials and Methods

### 2.1. Study Population

The KNHANES is a large-scale cross-sectional study based on the National Health Promotion Act. It is conducted to produce national statistics through a survey of disease status, health-related awareness and attitudes, and food and nutrition status [29]. The KNHANES was conducted at 3-year intervals from 1998 (1st survey) to 2005 (3rd survey); however, from the 4th survey onwards (2007–2009), the system was revised to include annual surveys. At present, the 8th KNHANES (2019–2021) is being conducted [30]. In this study, we analyzed data from 2014 to 2018, as these databases contain information on the main outcome variable, handgrip strength. Among a total of 39,199 participants, those aged <65 years (*n* = 31,109), those with missing values for amino acid intake or handgrip strength (*n* = 1895), those diagnosed with cancer or cardiovascular disease at the time of the survey (*n* = 1243), and those whose daily energy consumption was <500 kcal or >5000 kcal (*n* = 100) were excluded. Thus, 4852 participants were included in the analysis.

All data in this study were collected after obtaining written consent from each participant. The KNHANES 2014 was conducted with the approval of the Institutional Review Board (IRB) of the Korea Disease Control and Prevention Agency (KDCA), while the KNHANES 2015–2017 was conducted without the need for ethical review based on the opinion of the IRB and Bioethics and Safety Act, as the survey was performed for the purpose of national public welfare [31]. However, since 2018, the review procedure was resumed considering the collection of human-derived materials and provision of raw data to a third party; hence, the surveys were performed after IRB approval (approval number: 2013-12EXP-03-5C, 2018-01-03-P-A) [31].

### 2.2. Demographic and Lifestyle Information

Data on the participants’ age, sex, household income, and education level were collected through interviews conducted by a trained investigator, while data on physical activity, alcohol consumption, and smoking status were collected through self-reported questionnaires. Household income was categorized into quartiles according to the monthly average household equalization income. Education level was categorized as “less than high school graduation” and “high school graduation or higher” based on the distribution observed in the study population. With regard to smoking status, the participants were divided into “current smokers” and “non-smokers,” and the participants were also classified as “drinkers” and “non-drinkers” based on alcohol consumption in the past year. Regarding the use of dietary supplements, the participants were divided into “users” and “non-users” based on their response to the item, “Use of a dietary supplement for ≥2 weeks in the past year.” To calculate the physical activity level, metabolic equivalent tasks (METs-h/week) were calculated based on the number of days and hours of intense, moderate, or walking physical activity by assigning a weighted value to each exercise intensity [32] and categorized into tertiles.

### 2.3. Anthropometry and Dietary Information

Anthropometric measurements were taken by trained staff according to the “KNHANES Health Examination Guidelines” [33]. Bodyweight and height were measured in a standing upright position after the participants had removed their clothes, socks, and shoes, and changed into the provided gown. Body mass index (BMI) was calculated by dividing weight (kg) by the square of the height (m^2^). The World Health Organization criteria for Asian populations were used to identify participants with underweight/normal weight (BMI < 23 kg/m^2^), overweight (23 ≤ BMI < 25 kg/m^2^), and obesity (BMI ≥ 25 kg/m^2^) [34].

Food and nutrient intake levels were estimated using a 24-h recall, conducted through a direct interview with the participants regarding the foods and beverages consumed on the day before the survey date. To increase the accuracy of the collected dietary intake data and to aid the participant’s recall, a set of supplementary tools, including food models, containers, measuring cups, measuring spoons, and a 30-cm ruler, were used.

An amino acid database was established to determine the level of BCAA intake. All food items consumed by the participants were screened using 24-h recall data from the KNHANES 2014–2018. Subsequently, an amino acid content database was constructed using the 9th revision of the Korean Standard Food Composition Table, published by the Rural Development Administration [35], and the Computer-Aided Nutritional Analysis Program 5.0, developed by the Korean Nutrition Society [36]. If the same food items were processed or prepared differently (boiled, raw, baked, or dried), they were classified into one food item category by applying moisture conversion factors developed by KDCA [37]. If there was no available product brand name for a processed food item, the commonly used name was applied. For any data without information on the country of origin (domestic or imported) or the specific cut or part of the food item (e.g., fore shank, hind shank, center of the heel shank, and heel shank), the general food category was selected.

### 2.4. Assessment of the Handgrip Strength

Handgrip strength was measured as part of the KNHANES 2014–2018 [31]. Trained staff explained to the participants the measurement procedures and methods related to the use of the digital grip strength dynamometer (T.K.K 5401, Takei Scientific Instruments Co.,Ltd., Niigata City, Japan); measurements were taken three times for each hand in alternation, starting with the dominant hand [33]. In addition, the participants were given 60 sec to rest after each round of measurements of both hands, and at each round, the handle position and individual’s posture were verified for accuracy [33]. Participants were excluded from the handgrip strength measurement if they were found to have a functional constraint, such as a history of wrist surgery in the past 3 months or a complaint of discomfort during the visual examination and interview [33]. In this study, the mean of the triplicate measurements obtained using the dominant hand was calculated, and for participants who reported frequent use of both hands, the overall mean value for both hands was calculated.

### 2.5. Statistical Analyses

To determine the main food groups that contribute to BCAA intake, all food items were classified into 18 food groups according to the criteria of the KNHANES [31]. The food groups included grains and grain products, potatoes, sugars, and related products, legumes and legume products, nuts and seeds, vegetables, mushrooms, fruits, meat and meat products, eggs, fish and shellfish, seaweed, milk and dairy products, oils, beverages, seasoning, cooked/processed foods, and other products. A complex sample design was adopted for a comprehensive analysis of the KNHANES data [29]. All related parameters were considered in the analysis; they included “weight” to increase the accuracy of the estimation and representativeness of the population, “strata” to integrate the design layer for the estimation of variance, and the primary sampling unit, which was the survey district from where the samples were extracted [29]. The characteristics of the participants were presented as frequencies and percentages for categorical variables and as means and standard errors for continuous variables. Multivariable regression analysis was performed to examine the association between BCAA intake and handgrip strength. Potential confounding factors were selected through a comprehensive review of the preliminary analysis and included risk factors associated with handgrip strength and factors associated with BCAA intake [38,39,40,41,42]. The potential effect modification was examined using multiplicative terms of the statistical model, and no significant effect modifier was observed. All statistical analyses were performed using the Statistical Analysis System version 9.4 (SAS Institute, Cary, NC, USA), and statistical significance was tested based on a significance level of α = 0.05.

## 3. Results

The general characteristics of the participants according to BCAA intake levels are shown in Table 1. The median of BCAA intake for quartiles 1, 2, 3, and 4 was 3.18 g/d, 5.03 g/d, 7.04 g/d, and 10.69 g/d, respectively; the average age was 74.18, 73.17, 72.18, and 71.23 years, respectively, and the proportion of women was 74.8%, 62.3%, 51.9%, and 35.9%, respectively. The proportion of participants with a high physical activity level and dietary supplement use ranged from 25.1% to 40.7% and from 41.0% to 54.6% across the BCAA intake quartiles, respectively. The proportion of alcohol drinkers was 37.9%, 49.1%, 55.1%, and 65.3% and that of current smokers was 7.9%, 8.9%, 9.3%, and 11.3% in quartiles 1, 2, 3, and 4, respectively.

Table 2 presents the list of food groups that contributed to the intake of total BCAAs, isoleucine, leucine, and valine. The top five food groups were grains, meat, legumes, fish and shellfish, and vegetables. Grains contributed the most, while vegetables contributed the least to the intake of total BCAAs, isoleucine, and leucine. In the case of valine, grains contributed the most, whereas fish and shellfish contributed the least.

Participants were divided into quartiles according to their total BCAA intake (1st quartile group: lowest intake; 4th quartile group: highest intake), and the proportions of BCAAs derived from the various food groups were compared between the groups (Table 3). In the 1st quartile group, the proportion of BCAAs derived from grains and grain products and that derived from vegetables was the highest; these proportions decreased concurrently with an increase in total BCAA intake (all *p* for trend < 0.001). The proportions of BCAAs derived from meat and meat products, fish and shellfish, and other food groups tended to be higher for participants with a higher total BCAA intake (all *p* for trend < 0.001). The proportion of BCAAs derived from legumes and legume products did not differ between the groups.

Table 4 shows the association between BCAA intake and handgrip strength. After adjusting for age, sex, and total energy intake (Model 1), significant positive linear trends were observed between handgrip strength and an increased intake of BCAAs (all *p* for trend < 0.001), except in the case of valine. In the fully adjusted model (Model 3), these associations were attenuated, and the association between leucine intake and handgrip strength alone remained significant (*p* for trend = 0.03).

Table 5 presents the associations between handgrip strength and BCAA intake derived from grains and grain products, and non-grain food groups. In Models 1 and 2, a negative association was observed between handgrip strength and the intake of BCAAs from grains and grain products (all *p* for trend < 0.05). In the fully adjusted model (Model 3), this significant negative association was maintained for total BCAAs and valine (both *p* for trend = 0.03), while marginal significance was observed for isoleucine (*p* for trend = 0.05) and leucine (*p* for trend = 0.047). Furthermore, significant positive linearity was observed between handgrip strength and an increased intake of isoleucine, leucine, valine, and total BCAAs from non-grain food sources (all *p* for trend < 0.05).

## 4. Discussion

Although Korean older adults mainly obtained BCAAs from grains and grain products, those with a high total BCAA intake tended to obtain BCAAs from a variety of food sources, while those with a low total BCAA intake tended to obtain BCAAs from grains and vegetables. In addition, BCAAs obtained from non-grain sources were associated with high handgrip strength, whereas those derived from grains were associated with low handgrip strength.

Among plant-based protein sources, rice is a staple food for Koreans, providing approximately 60% of the starch-based calories in this population [43,44]. In our study, grains and grain products were the major sources of total BCAA intake in Korean older adults. Similarly, grains were found to be the highest contributors of protein intake in adults aged ≥60 years in a previous study that used data from the KNHANES 2013–2014 [27]. However, we found that grains were the biggest source of BCAAs in participants with relatively low BCAA intake levels, while those with higher intake levels tended to obtain BCAAs from a variety of sources. This indicates that consuming a varied diet (especially non-grain foods) is important for increasing the total BCAA intake in the Korean older population. Furthermore, we observed a significant negative association between handgrip strength and BCAAs derived from grains, whereas BCAAs derived from non-grain food groups had a positive linear association with handgrip strength. Plant proteins, such as grains, contain relatively fewer essential amino acids (especially leucine) than animal proteins [45]. When the concentrations of essential amino acids, including BCAAs, are insufficient in the body, other amino acids cannot be utilized efficiently and may be oxidized, resulting in an adverse effect on protein synthesis [45,46]. In the Health, Aging, and Body Composition Study, changes in lean body mass were compared according to the intake of different protein sources among 3075 older individuals aged 70–79 years, and a significant positive association was observed only with respect to animal proteins, unlike for plant proteins [47]. In addition, loss of grip strength declined linearly with increasing intake of total and animal protein, but not with an increase in plant protein intake, in older adults in Framingham, USA [48]. It is known that Korean older adults rely heavily on non-animal proteins [49,50], leading to a low intake of animal proteins. Thus, it is necessary to emphasize the importance of protein intake from a variety of food sources in the Korean older population.

In the present study, a high leucine intake was associated with an increase in handgrip strength, consistent with the results of previous studies in older populations. In a cross-sectional study conducted in Indonesia, a significant positive correlation was observed between leucine intake and handgrip strength in older people aged 60–69 years [25]. In an 8-week clinical study conducted in Japan, a significant time-dependent increase in handgrip strength was observed in the intervention group of older individuals aged ≥ 65 years who were advised to take leucine supplements [26]. Leucine is known to directly regulate the mammalian target of rapamycin complex 1 (mTORC1), which activates protein synthesis and inhibits protein degradation [51]. The mechanism is as follows: leucine enters the body through dietary intake and binds with sestrin2, leading to its detachment from guanosine triphosphatase activating protein toward Rags (GATOR)-2 [52]. The independent GATOR2 complex then negatively regulates GATOR1 and subsequently drives the Rag-Regulator system to move mTORC1 to the lysosomal surface [52]. Activated mTORC1, after attachment to the surface of lysosomes, phosphorylates eIF4E-binding protein 1 and S6 kinase 1, which are translation initiation factors involved in the initiation of mRNA translation [52]. Another possible explanation is the effect of β-hydroxy-β-methylbutyrate (HMB). HMB is a metabolite of leucine and is known to decrease protein catabolism by downregulating the ubiquitin–proteasome pathway and reducing caspase activity [53]. The ubiquitin–proteasome pathway is one of the main mechanisms of protein degradation [54], while caspases are involved in executing apoptosis [55]. Recent studies have reported a positive effect of HMB on muscle strength in older people. In a clinical trial conducted in Italy, 80 older women aged ≥65 years were randomly assigned to the supplementation or control group [56]. After 8 weeks of follow-up, a significant increase in handgrip strength was observed in the HMB supplementation group compared to that in the control group [56]. In addition, in healthy older men, muscle contraction showed a significant increase after 6 weeks compared with baseline measurements [57].

This study has some limitations. The KNHANES is a cross-sectional survey; therefore, the cause–effect relationship between the intake of BCAAs and handgrip strength could not be established. In addition, 24-h recall data were used to construct the dietary amino acid database, and these data might not have reflected the usual dietary intake. However, a trained interviewer conducted the survey using standardized protocols, and participants with extreme levels of energy intake were excluded to minimize the likelihood of error. Information regarding dietary supplements was not obtained in the KNHANES; hence, the intake of amino acids through dietary supplements could not be considered. Finally, the 9th revision of the Korean Standard Food Composition used for constructing the database did not provide the amino acid content of certain foods. Therefore, it is possible that dietary intake among the participants was underestimated. As a complementary measure, alternative protocols were established, such as examining the food composition or applying the moisture coefficient, and multiple food composition databases were used to minimize the number of missing foods. Despite these limitations, the significance of this study is that it is the first to construct a database of dietary amino acids consumed by Korean older adults to investigate the association between the intake of BCAAs and handgrip strength. The findings of this study are anticipated to be used as basic scientific data for devising nutrition management plans for muscular health in older people.

## 5. Conclusions

The association between BCAA intake and handgrip strength was examined among Korean older adults aged ≥65 years, and a significant positive association was observed between leucine intake and handgrip strength. In addition, a significant positive linear association was observed between handgrip strength and increased BCAA intake from non-grain food sources. Thus, the importance of BCAA intake (especially leucine intake) through a variety of food sources should be considered to improve muscle strength in Korean older adults. The causal relationship between BCAA intake and handgrip strength should be investigated in a follow-up large-scale prospective cohort study that considers dietary intake data with minimum measurement errors (such as 24-h recall data for ≥3 days) as well as the intake of dietary supplements.

## Figures and Tables

**Table 1:** Characteristics of participants according to BCAA intake levels.

	BCAA Intake Levels (g/d)			
	Q1	Q2	Q3	Q4
*n*	1213	1213	1213	1213
BCAA intake, range (median)	0.44–4.13 (3.18)	4.14–5.95 (5.03)	5.96–8.43 (7.04)	8.44–45.70 (10.69)
Isoleucine intake	0.70 ± 0.01	1.17 ± 0.01	1.68 ± 0.01	2.82 ± 0.01
Leucine intake	1.41 ± 0.02	2.31 ± 0.02	3.24 ± 0.02	5.37 ± 0.02
Valine intake	0.96 ± 0.02	1.55 ± 0.02	2.18 ± 0.02	3.53 ± 0.02
Total energy intake (kcal)	1088.26 ± 13.81	1461.26 ± 13.81	1773.66 ± 13.81	2389.64 ± 13.81
Handgrip strength (kg)	20.02 ± 0.24	22.64 ± 0.24	24.80 ± 0.24	27.77 ± 0.24
Age (years)	74.18 ± 0.14	73.17 ± 0.14	72.18 ± 0.14	71.23 ± 0.14
Sex				
Men	306 (25.2)	457 (37.7)	584 (48.2)	777 (64.1)
Women	907 (74.8)	756 (62.3)	629 (51.9)	436 (35.9)
Education level				
Less than high school graduation	940 (87.0)	876 (79.5)	797 (70.7)	653 (57.1)
High school graduation or higher	140 (13.0)	226 (20.5)	331 (29.3)	490 (42.9)
Household income				
Low	730 (60.4)	629 (52.3)	537 (44.6)	388 (32.2)
Mid-low	283 (23.4)	339 (28.2)	316 (26.3)	368 (30.5)
Mid-high	127 (10.5)	132 (11.0)	199 (16.5)	248 (20.6)
High	68 (5.6)	102 (8.5)	152 (12.6)	202 (16.8)
Physical activity level ^1^				
Low	444 (41.1)	363 (32.9)	339 (30.0)	276 (24.2)
Mid	365 (33.8)	397 (36.0)	404 (35.8)	401 (35.1)
High	271 (25.1)	342 (31.0)	386 (34.2)	464 (40.7)
Body mass index (kg/m^2^)				
<23	454 (37.8)	442 (36.7)	470 (38.8)	436 (36.0)
23–<25	301 (25.0)	332 (27.6)	292 (24.1)	343 (28.4)
≥25	447 (37.2)	429 (35.7)	448 (37.0)	431 (35.6)
Dietary supplement				
Non-users	716 (59.0)	614 (50.6)	613 (50.5)	550 (45.4)
Users	497 (41.0)	599 (49.4)	600 (49.5)	662 (54.6)
Alcohol consumption				
Non-drinkers	709 (62.1)	590 (50.9)	529 (44.9)	410 (34.7)
Drinkers	432 (37.9)	569 (49.1)	649 (55.1)	772 (65.3)
Smoking status				
Non-smokers	1051 (92.1)	1055 (91.1)	1064 (90.7)	1048 (88.7)
Current smokers	90 (7.9)	103 (8.9)	109 (9.3)	134 (11.3)

BCAA, branched-chain amino acid; Q, quartile. Values are expressed as the mean ± standard error or n (%). ^1^ The physical activity level was calculated in terms of metabolic equivalent tasks (METs-h/week) and categorized into tertiles.

**Table 2:** Food groups contributing to the intake of BCAAs among participants aged ≥65 years.

Food Groups	Contribution (%)	Intake (g/d) ^1^
BCAA		
Grains and grain products	42.58	2.53 ± 1.3
Meat and meat products	21.87	1.90 ± 2.3
Legumes and legume products	13.34	0.93 ± 1.1
Fish and shellfish	8.75	0.70 ± 1.4
Vegetables	8.56	0.52 ± 0.4
Other food groups ^2^	19.21	1.33 ± 1.4
Isoleucine		
Grains and grain products	39.84	0.55 ± 0.3
Meat and meat products	23.61	0.49 ± 0.6
Legumes and legume products	14.17	0.23 ± 0.3
Fish and shellfish	9.44	0.18 ± 0.4
Vegetables	8.96	0.13 ± 0.1
Other food groups ^2^	19.44	0.32 ± 0.3
Leucine		
Grains and grain products	43.02	1.17 ± 0.6
Meat and meat products	21.74	0.86 ± 1.0
Legumes and legume products	14.16	0.46 ± 0.5
Fish and shellfish	8.77	0.32 ± 0.6
Vegetables	8.04	0.22 ± 0.2
Other food groups ^2^	18.82	0.59 ± 0.6
Valine		
Grains and grain products	44.14	0.81 ± 0.4
Meat and meat products	20.78	0.55 ± 0.7
Legumes and legume products	11.61	0.24 ± 0.3
Vegetables	9.06	0.17 ± 0.1
Fish and shellfish	8.22	0.20 ± 0.4
Other food groups ^2^	19.58	0.42 ± 0.4

BCAA, branched-chain amino acids ^1^ Values are expressed as the mean ± standard error. ^2^ Other food groups include potatoes, sugars and related products, nuts and seeds, mushrooms, fruits, eggs, seaweed, milk and dairy products, oils, beverages, seasoning, cooked/processed foods, and other products.

**Table 3:** Average proportion of amino acid intake from different food groups according to the quartiles of BCAA intake.

	Q1	Q2	Q3	Q4	*p* for Trend
	*n* = 1213	*n* = 1213	*n* = 1213	*n* = 1213	
BCAA (g/d)					
Grains and grain products	61.63 ± 0.84	47.60 ± 0.61	36.96 ± 0.47	22.89 ± 0.56	<0.001
Meat and meat products	8.76 ± 0.89	18.18 ± 0.72	21.94 ± 0.69	31.23 ± 0.98	<0.001
Legumes and legume products	12.95 ± 0.60	12.65 ± 0.49	12.80 ± 0.41	14.37 ± 0.63	0.07
Fish and shellfish	4.13 ± 0.51	7.11 ± 0.42	9.59 ± 0.49	15.21 ± 0.80	<0.001
Vegetables	11.57 ± 0.31	9.26 ± 0.22	7.80 ± 0.18	5.67 ± 0.20	<0.001
Other food groups ^1^	14.86 ± 0.65	19.28 ± 0.58	21.61 ± 0.58	21.27 ± 0.66	<0.001
Isoleucine (g/d)					
Grains and grain products	60.26 ± 0.83	44.84 ± 0.58	33.93 ± 0.43	19.52 ± 0.51	<0.001
Meat and meat products	8.62 ± 0.91	18.89 ± 0.72	24.05 ± 0.70	34.08 ± 0.96	<0.001
Legumes and legume products	13.79 ± 0.63	13.90 ± 0.52	13.41 ± 0.44	14.87 ± 0.65	0.24
Fish and shellfish	4.38 ± 0.55	7.50 ± 0.43	10.46 ± 0.53	16.40 ± 0.83	<0.001
Vegetables	12.41 ± 0.33	9.79 ± 0.23	8.10 ± 0.18	5.63 ± 0.20	<0.001
Other food groups ^1^	15.44 ± 0.65	19.93 ± 0.60	21.66 ± 0.59	20.93 ± 0.65	<0.001
Leucine (g/d)					
Grains and grain products	61.83 ± 0.84	47.32 ± 0.61	37.68 ± 0.45	23.84 ± 0.57	<0.001
Meat and meat products	9.43 ± 0.90	17.61 ± 0.73	22.17 ± 0.68	30.92 ± 0.98	<0.001
Legumes and legume products	13.51 ± 0.64	13.55 ± 0.51	13.23 ± 0.41	15.84 ± 0.68	0.01
Fish and shellfish	4.19 ± 0.52	7.07 ± 0.42	9.56 ± 0.50	15.24 ± 0.79	<0.001
Vegetables	10.91 ± 0.31	8.80 ± 0.22	7.24 ± 0.17	5.23 ± 0.19	<0.001
Other food groups ^1^	14.77 ± 0.63	19.46 ± 0.61	21.03 ± 0.55	20.18 ± 0.66	<0.001
Valine (g/d)					
Grains and grain products	62.08 ± 0.84	49.68 ± 0.60	38.92 ± 0.49	24.61 ± 0.57	<0.001
Meat and meat products	8.42 ± 0.86	17.61 ± 0.69	20.55 ± 0.65	30.08 ± 0.96	<0.001
Legumes and legume products	12.42 ± 0.60	10.96 ± 0.42	10.92 ± 0.36	11.70 ± 0.57	0.82
Fish and shellfish	3.94 ± 0.51	6.76 ± 0.39	9.17 ± 0.46	14.08 ± 0.77	<0.001
Vegetables	11.78 ± 0.32	9.80 ± 0.21	8.39 ± 0.19	6.33 ± 0.21	<0.001
Other food groups ^1^	14.25 ± 0.68	18.54 ± 0.57	22.07 ± 0.58	23.74 ± 0.74	<0.001

Q, quartile; BCAA, branched-chain amino acid. Values are expressed as means ± standard errors, adjusted for age, sex, and total energy intake. ^1^ Other food groups include potatoes, sugars, and related products, nuts and seeds, mushrooms, fruits, eggs, seaweed, milk and dairy products, oils, beverages, seasoning, cooked/processed foods, and other products.

**Table 4:** Multivariable regression analysis between BCAA intake and handgrip strength among participants aged ≥65 years.

	Q1	Q2	Q3	Q4	*p* for Trend
	*n* = 1213	*n* = 1213	*n* = 1213	*n* = 1213	
		β-Coefficient ± SE	β-Coefficient ± SE	β-Coefficient ± SE	
BCAA (g/d)					
Model 1	ref	0.304 ± 0.270	0.694 ± 0.294	0.907 ± 0.359	0.01
Model 2	ref	0.131 ± 0.281	0.468 ± 0.297	0.585 ± 0.368	0.09
Model 3	ref	0.137 ± 0.288	0.572 ± 0.307	0.623 ± 0.379	0.07
Isoleucine (g/d)					
Model 1	ref	0.488 ± 0.265	0.653 ± 0.292	0.914 ± 0.351	0.02
Model 2	ref	0.295 ± 0.274	0.433 ± 0.296	0.612 ± 0.358	0.11
Model 3	ref	0.290 ± 0.280	0.551 ± 0.309	0.663 ± 0.371	0.08
Leucine (g/d)					
Model 1	ref	0.325 ± 0.273	0.729 ± 0.297	1.086 ± 0.362	0.002
Model 2	ref	0.140 ± 0.280	0.539 ± 0.300	0.749 ± 0.372	0.03
Model 3	ref	0.192 ± 0.287	0.625 ± 0.309	0.796 ± 0.381	0.03
Valine (g/d)					
Model 1	ref	0.281 ± 0.278	0.734 ± 0.296	0.644 ± 0.380	0.08
Model 2	ref	0.110 ± 0.287	0.538 ± 0.298	0.373 ± 0.390	0.28
Model 3	ref	0.089 ± 0.296	0.621 ± 0.306	0.361 ± 0.406	0.29

Q, quartile; SE, standard error; BCAA, branched-chain amino acid. Model 1: Adjusted for age, sex, and total energy intake; Model 2: Additionally adjusted for smoking status, alcohol consumption, household income, and education level; Model 3: Additionally adjusted for body mass index, physical activity level, dietary supplement use, and type 2 diabetes.

**Table 5:** Multivariable regression analysis between BCAA intake and handgrip strength among participants aged ≥65 years according to grains and non-grain food groups.

	Q1	Q2	Q3	Q4	*p* for Trend
		β-Coefficient ± SE	β-Coefficient ± SE	β-Coefficient ± SE	
Grains and grain products					
BCAA (g/d)					
Model 1	ref	−0.405 ± 0.255	−0.825 ± 0.275	−1.247 ± 0.322	<0.001
Model 2	ref	−0.262 ± 0.253	−0.656 ± 0.279	−0.779 ± 0.326	0.01
Model 3	ref	−0.109 ± 0.256	−0.515 ± 0.283	−0.641 ± 0.326	0.03
Isoleucine (g/d)					
Model 1	ref	−0.279 ± 0.253	−0.763 ± 0.273	−1.030 ± 0.318	<0.001
Model 2	ref	−0.175 ± 0.255	−0.656 ± 0.279	−0.618 ± 0.321	0.03
Model 3	ref	−0.045 ± 0.257	−0.543 ± 0.282	−0.534 ± 0.320	0.05
Leucine (g/d)					
Model 1	ref	−0.405 ± 0.252	−0.885 ± 0.272	−1.120 ± 0.332	<0.001
Model 2	ref	−0.287 ± 0.252	−0.769 ± 0.275	−0.695 ± 0.338	0.03
Model 3	ref	−0.121 ± 0.256	−0.648 ± 0.280	−0.593 ± 0.339	0.047
Valine (g/d)					
Model 1	ref	−0.384 ± 0.256	−0.859 ± 0.275	−1.271 ± 0.325	<0.001
Model 2	ref	−0.331 ± 0.254	−0.686 ± 0.280	−0.828 ± 0.331	0.01
Model 3	ref	−0.186 ± 0.257	−0.557 ± 0.286	−0.668 ± 0.331	0.03
Non-grain food groups ^1^					
BCAA (g/d)					
Model 1	ref	0.650 ± 0.263	0.632 ± 0.283	1.203 ± 0.314	<0.001
Model 2	ref	0.479 ± 0.267	0.216 ± 0.296	0.805 ± 0.335	0.03
Model 3	ref	0.419 ± 0.278	0.315 ± 0.302	0.832 ± 0.339	0.02
Isoleucine (g/d)					
Model 1	ref	0.722 ± 0.261	0.619 ± 0.282	1.262 ± 0.322	<0.001
Model 2	ref	0.582 ± 0.268	0.209 ± 0.294	0.845 ± 0.342	0.04
Model 3	ref	0.543 ± 0.278	0.298 ± 0.302	0.850 ± 0.347	0.03
Leucine (g/d)					
Model 1	ref	0.547 ± 0.265	0.686 ± 0.279	1.260 ± 0.319	<0.001
Model 2	ref	0.402 ± 0.268	0.355 ± 0.291	0.843 ± 0.337	0.02
Model 3	ref	0.334 ± 0.276	0.413 ± 0.295	0.872 ± 0.339	0.01
Valine (g/d)					
Model 1	ref	0.666 ± 0.265	0.614 ± 0.286	1.287 ± 0.321	<0.001
Model 2	ref	0.484 ± 0.268	0.228 ± 0.298	0.850 ± 0.341	0.03
Model 3	ref	0.433 ± 0.278	0.302 ± 0.305	0.896 ± 0.349	0.02

BCAA, branched-chain amino acid; Q, quartile; SE, standard error. ^1^ Non-grain food sources include meat and meat products, legumes and legume products, fish and shellfish, vegetables, and other food groups (potatoes, sugars, and related products, nuts and seeds, mushrooms, fruits, eggs, seaweed, milk and dairy products, oils, beverages, seasoning, cooked/processed foods, and other products). Model 1: Adjusted for age, sex, and total energy intake; Model 2: Additionally adjusted for smoking status, alcohol consumption, household income, and education level; Model 3: additionally adjusted for body mass index, physical activity, dietary supplement use, and type 2 diabetes.
